# Active Adjustment of the Subreflector Shape for the Large Dual-Reflector Antenna

**DOI:** 10.3390/mi14101893

**Published:** 2023-09-30

**Authors:** Binbin Xiang, Tianxiang Zheng, Wei Wang, Peiyuan Lian, Guljaina Kazezkhan, Jianping Zhou, Kai Li

**Affiliations:** 1 School of Mechanical Engineering, Xinjiang University, Urumqi 830017, China; 2 School of Mechano-Electronic Engineering, Xidian University, Xi’an 710071, China; 3 Xinjiang Astronomical Observatory, Chinese Academy of Sciences, Urumqi 830011, China

**Keywords:** dual-reflector antenna, structural deformation, wavefront error, subreflector, active adjustment

## Abstract

A shape adjustment method for subreflectors based on minimizing the residual wavefront error of the large dual-reflector antenna is presented. This method is used to compensate for the antenna structural deformation caused by environment loading. The shape of the subreflector is adjusted using actuators fixed under the panels. The shape adjustment response function for the subreflector shape and the actuators’ adjustment amount is established, which is based on the inverse distance weighting function, and then the control function of the subreflector shape is obtained. The actuators’ adjustment amount can be calculated using the least squares matrix transformation with the minimum residual wavefront error. Analysis of the experiment’s results shows the residual wavefront error and primary aberration are greatly reduced under different elevation angles, and the effectiveness of the proposed method is verified.

## 1. Introduction

Radio telescopes, used to receive electromagnetic waves from the universe, usually employ a large dual-reflector antenna as the signal-receiving device. These antennas can generally be enhanced by increasing the area of the main reflector and improving the surface accuracy to improve the receiving capability. However, environmental factors such as gravity, temperature, and wind can cause structural deformation of the large antenna. This deformation leads to distortion of the primary reflector surface and displacement of the subreflector, resulting in a loss of antenna efficiency. Additionally, structural resonance, electromagnetic interference, and other factors can also reduce the antenna’s receiving performance. One way to correct the influence of structural deformation is through the active adjustment of the subreflector. Therefore, an accurate adjustment method is needed to compensate for the structural error and improve the antenna’s electromagnetic performance.

For the dual-reflector antenna, the structural deformation error can be compensated by adjusting the position of the subreflector. Multiple degrees of freedom can be used to actively correct large-scale wavefront errors, specifically low-order spatial errors, in the aperture plane through the rigid body displacement movement [1]. The deformation errors caused by the gravity are systematic errors and consistent over time under the same load conditions. A mathematical model of the active adjustment can be established to adjust and correct the subreflector position in real time at various elevation angles.

The author of this research discussed the compensation of low-order residual errors of the subreflector through the rigid body displacement of five degrees of freedom and deduced the relationship between the rigid body displacement and the relative gain by analyzing the wavefront error characteristics caused by the structural deformation of the reflector.

In reference [2], a technique for describing the surface of a double-biased reflector based on a finite element was presented. This technique involved assuming a fixed shape for the primary reflector and achieving comprehensive adjustment through a deformable surface of the subreflector. The consideration of mechanical properties of the deformable surface and the influence of control points were also included in the analysis.

To correct the thermal distortion of the primary reflector, an electroformed Nickel deformable shell was used on the subreflector [3]. This deformable reflector can be operated in adaptive mode. Reference [4] describes the design of a nine-point actuator deformation mirror specifically for space cameras aimed at correcting low-order aberrations.

Based on the physical optics, reference [5] proposed a new method to determine the panel adjustment value from the far field pattern. This involved constructing a linear system between the actuator adjustment amount and the far field pattern, which was constructed by deducing the influence of the panel displacement on the radiation field value. In order to optimize the algorithm for the radio telescope and improve the measurement accuracy, reference [6] proposed an accuracy estimation of least square parameters. In the study [7,8,9], researchers analyze the influence of actuator adjustment on specular error based on active optics, obtain the correspondence formula, and verify it using the finite element method. Reference [10] proposes to compensate for structural deformation by using angle sensors on adjacent panels to obtain angular transformations. The goal of [11] is to investigate how panel-setting errors affect electrical performance and to establish practical error budgets; an approximate expression for the error-transformation matrix (ETM) between panel-setting errors and aperture errors is derived. Reference [12] improve the compensation efficiency of panels by improving panel strength and adding additional test machines. An optimization method for active mirror elements was proposed [13,14], which coupled the minimization of the gravity influence function with the optimization of the combinatorial actuator influence function into a low-order aberration. Reference [15] demonstrated the feasibility of electronically controlled reconfiguration of a mesh reflector antenna. This method can also be applied to the control of the surface shape of the subreflector. References [16,17] studied the effect of panel adjustment error on the electrical performance of rigid panel and deduced the approximate expression of panel adjustment error and aperture phase error for the three-ring reflector.

Currently, numerous studies have been conducted on the compensation of structural deformation, covering topics such as the structure, algorithm, material, and optical properties. These investigations aim to enhance the receiving performance of antennas, either directly or indirectly, by addressing different aspects. However, when considering aberration analysis, the aforementioned methods only focus on low-order optimization of the antenna. Consequently, there remain residual astigmatism, coma, and spherical aberrations (wavefront errors) due to the incomplete correction of the wavefront error of the aperture plane caused by the rigid motion of the subreflector using various approaches. The residual wavefront error can be further corrected by the shape adjustment to reduce the influence of the wavefront error (phase). It is necessary to further research the adjustment method of the subreflector shape.

In this paper, the correction of residual wavefront aberrations, such as spherical aberration, astigmatism, and coma, will be achieved through the adjustment of the subreflector shape. The objective is to minimize the residual wavefront error. A geometric expression relating the adjustment amounts of the subreflector actuators to the wave path difference is derived. The least square method will be utilized to solve the expression.

Section 2 discusses the influence of the surface errors on the wave path difference of the subreflector. Section 3 proposes a shape adjustment method to determine the movements of the subreflector actuators. Section 4 describes the effectiveness of the method through the simulation experiments. 

## 2. Influence of the Subreflector Surface Error

In order to improve antenna electromagnetic performance by compensating for residual small-scale errors and correcting the primary wavefront aberration caused by structural deformation, the study focuses on the shape adjustment of the subreflector using actuators. First, the rigid body motion of the subreflector in the five degrees of freedom, is used to correct the primary wavefront aberration by compensating for the position deviation caused by structural deformation. The adjustment method of the subreflector in the five degrees of freedom described in detail in [1], is employed for this purpose. However, despite the use of this method, there are still residual small-scale errors that need to be addressed. Therefore, in this paper, the shape adjustment of the subreflector using actuators is investigated as a means to compensate for these residual errors and enhance the antenna’s electromagnetic performance.

**Figure 1:**
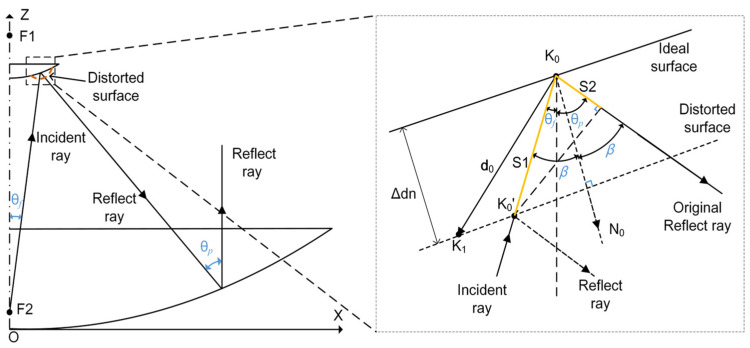
The effect of surface error on wave path difference.

The distortion of the subreflector surface alters the path length of radio wave propagation, resulting in a difference in wave path on the aperture plane. As Figure 1 shows, the distortion of the subreflector leads to the point K_0_ on the ideal surface into point K_0_, and the path length of the incident ray changes S1 and the path length of the reflected ray changes S2, and *β* is the half angle between the incident ray and reflected ray, Δ*d_n_* is the normal displacement of the planes. The wave path difference can be expressed as

(1) $$\delta_{s}=S1+S2=2{\vec{d}}_{0}\cdot {\vec{N}}_{0}\cdot \cos[(\theta_{f}+\theta_{P})/2]$$

where *d*_0_ and *N*_0_ are the displacement vectors and normal vectors of the subreflector, respectively.

Here, we assume that the adjustment mechanisms of the main reflector and subreflector have already made initial corrections to large-scale errors and only made secondary corrections for small-scale errors on the subreflector. From the perspective of geometrical optics, it can be concluded that there are still residual wavefront errors (wave path difference) resulting from surface shape distortion after the subreflector is adjusted in the direction of five degrees of freedom. Thus, to enhance the antenna efficiency, it is crucial to minimize residual wavefront errors via shape adjustment.

## 3. Shape Adjustment of the Subreflector

The subreflector is actively adjusted by controlling the length of the actuators, which serve as the panel adjustment mechanism. It is crucial to determine the appropriate adjustment amount for the subreflector actuators in order to achieve optimal antenna performance. In this paper we propose a method of the surface shape matrix transformation with the minimum residual wave path difference to obtain subreflector actuators length. The adjustment vector of the actuators is calculated by the matrix transformation between the actuators influence matrix and the surface distortion matrix based on the least squares method, and the goal is the minimum residual wave path difference after actuators is adjusted.

### 3.1. Actuators Influence Matrix

A key aspect for determining the actuator’s adjustment amount is understanding the influence of actuator’s adjustment on the subreflector surface shape. Therefore, it is necessary to obtain the shape adjustment response function (SAF), which represents the relationship between the movement of the subreflector adjustment point and the surface shape error.

**Figure 2:**
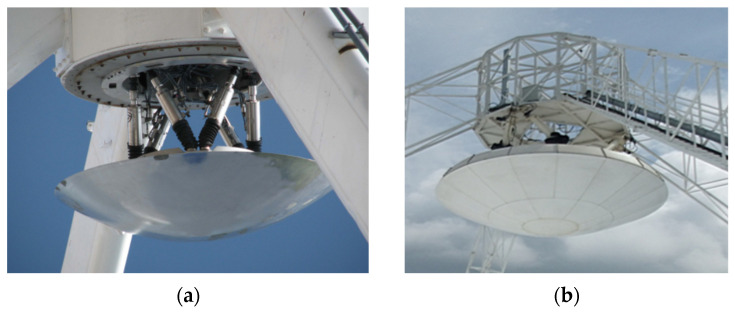
Subreflector of the dual-reflector antenna (**a**) integrated subreflector; (**b**) segmented subreflector.

There are two types of subreflector structural forms, namely, the integrated subreflector and the segmented subreflector, as shown in Figure 2. The integrated subreflector is made up of a single panel, while the segmented subreflector consists of multiple segment panels. When adjusting an actuator, the surface shape of the integrated subreflector will be affected across its entirety, as well as the surface shape of the segmented subreflector within a specific segment panel range. The influence of an actuator on surface shape can be calculated by the SAF and will be described below.

**Figure 3:**
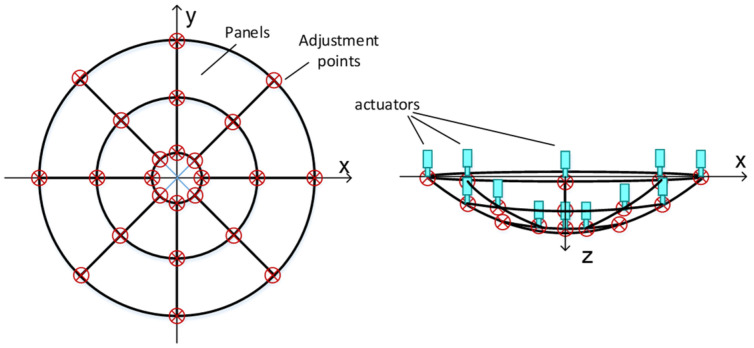
A deformable subreflector with the actuator’s adjustment.

First, we will study the segment panel with corner points actuated and then extend to the integrated subreflector. As shown in Figure 3, the apparatus that can actively control the shape of the subreflector consists of splicing panels and the adjustment mechanism located at the corner of each panel. Each actuator will affect adjacent two or four panels, and the entire secondary reflector error will be corrected by obtaining the adjustment amount of each actuator. Therefore, the elongation of the actuator is the main factor in correcting the deformation of the secondary surface. 

A function is required to describe the surface shape parameters, including reflector shape, actuator adjustment amount, actual and target shape, and actuator length. The change of the subreflector surface caused by actuators can be expressed as

(2) δ(x,y)=V(u)

where *δ* (*x*, *y*) is the normal displacement at point (*x*, *y*) on the surface, *V*(·) is the response function of the actuators (SAF), and *u* is the adjustment length of the actuators. Based on the SAF, the panel shape can be adjusted, and then the wavefront error of the subreflector can be corrected.

**Figure 4:**
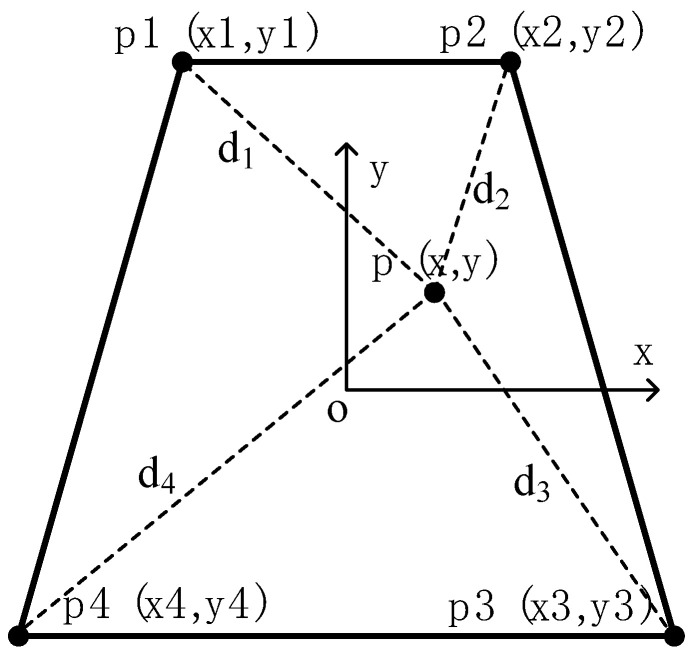
Schematic diagram of trapezoidal panel.

The panel for the active adjustment system of the integrated subreflector is approximately trapezoidal in shape and supported by four actuators at the corners, as shown in Figure 4. However, the four adjustment points for support create an over-constraint plane causing local deformation. It should be noted that the overall deformation is the cumulative effect of all the actuator adjustments, following the superposition principle. The deformation of the entire panel is the superposition of panel distortion caused by the movement of a single actuator, as the deformation of a single panel is minimal. The subreflector shape’s overall deformation can be expressed as:

(3) $$\delta (x,y)=\sum_{i=1}^{n}\delta_{i}\left(x,y\right)$$

where $\delta_{i}\left(x,y\right)$ is the shape deviation caused by the movement of the *i*th actuator in the surface point (*x*, *y*), and *n* is the number of actuators. A single panel is controlled by four actuators.

**Figure 5:**
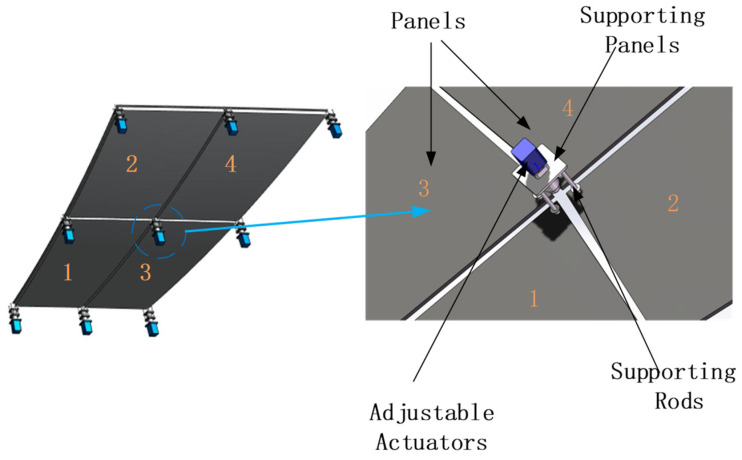
Panels and actuators supporting structure (shared actuators).

The SAF can be obtained from two aspects: the deformation state of the panels is obtained by measuring the motion of the actuator with the measuring equipment, and the numerical simulation of the panel with the finite element method can truly reflect the SAF according to the accuracy of the model. As shown in Figure 5, the shared actuator support structure refers to an actuator that simultaneously supports the corners of four adjacent panels. The situation can be approximately equivalent to the continuous shape control of the reflector by an actuator. It can be assumed that the actuator adjustment has been obtained, and the surface shape of the whole reflector can be calculated by using a certain two-dimensional interpolation function.

The subreflector panel is typically made of aluminum skin and a steel backbar frame. The primary function of the dorsal tendons is to provide stiffness, while the skin is mainly used to emit electromagnetic waves. The panel can serve as a plane bending unit, and the fan-shaped panel can be simplified into a trapezoidal shape, as shown in Figure 5. For the active optics technology in an optical telescope, the Zernike polynomial can be used to represent and analyze the surface error of optical parts and the wave aberration of the system conveniently, and the residual surface shape error of the reflector and the SAF of each actuator can be simplified by Zernike polynomial. The inverse distance weighting function can be used as the displacement interpolation function of the bending element. The basic idea of this method is the distance from the estimated grid point. It is assumed that the *N* points closest to the grid point have an influence on it, then the influence of these *N* points on the grid point is inversely proportional to the distance. Therefore, the displacement of each point p on the panel is given by

(4) $$\delta (x,y)=\sum_{i=1}^{4}\lambda_{i}(x,y)\cdot u_{i}$$

where *u_i_* is the displacement of points $p_{1}$, $p_{2}$, $p_{3}$ and $p_{4}$ on the panel, respectively, as shown in Figure 4, $\lambda_{i}$ is the inverse distance coordinate of *p*(*x*, *y*), and can be defined

(5) $$\lambda_{i}(x,y)=\frac{\frac{1}{d_{i}^{\mu }}}{\sum_{i=1}^{N}\frac{1}{d_{i}^{\mu }}}$$

where *d_i_* is the space distance between the point p(*x*, *y*) in the panel surface and the known adjustment point *p_i_*, $d_{i}=\sqrt{{\left(x-x_{i}\right)}^{2}+{\left(y-y_{i}\right)}^{2}}$, *μ* is a weighted index parameter used to control the influence range of data point values (*μ > 0*) [18,19]. The *μ* value can be determined by fitting the panel shape data from experimental or finite element analysis using the optimization method as

(6) $$\begin{array}{c} \mathrm{find}:\;\mu \\ \min:\;\left\|\delta -\hat{\delta }\right\| \end{array}$$

The objective of optimization is to minimize panel shape deviation, represented by the rms value. This paper uses finite element analysis to determine the deformation of the panel, which is influenced by its size and stiffness. The shape adjustment response function of the segment panel can be expressed as

(7) $$\left[\begin{array}{c} \delta_{1}\\ \delta_{2}\\ \vdots \\ \delta_{m} \end{array}\right]=\left[\begin{array}{cccc} \lambda_{11} & \lambda_{12} & \lambda_{13} & \lambda_{14}\\ \lambda_{21} & \lambda_{22} & \lambda_{23} & \lambda_{24}\\ \vdots & \vdots & \vdots & \vdots \\ \lambda_{m1} & \lambda_{m2} & \lambda_{m3} & \lambda_{m4} \end{array}\right]\left[\begin{array}{c} u_{1}\\ u_{2}\\ u_{3}\\ u_{4} \end{array}\right]\;\hat{s}=\hat{V}\cdot \hat{u}$$

where $\hat{s}$ is the displacement vector surface, $\hat{V}$ is the actuators influence matrix of the segment panel, and $\hat{u}$ is the adjustment length matrix of the 4 actuators, $\hat{s}={\left[\begin{array}{ccc} \delta_{1} & \begin{array}{ccc} \cdots & \delta_{j} & \cdots \end{array} & \delta_{m} \end{array}\right]}^{T}$, $\delta_{j}=\delta (x_{j},y_{j})=\sum_{i=1}^{4}\lambda_{ij}\cdot u_{i}\;$, $\lambda_{ij}=\lambda_{i}(x_{j},y_{j})$.

The influence of the actuator’s adjustment of the segment panel can be expressed by Equation (7) and extended to the integrated subreflector. The whole surface shape of the subreflector can be determined by

(8) $$\left[\begin{array}{c} \delta_{1}\\ \vdots \\ \delta_{j}\\ \vdots \\ \delta_{m} \end{array}\right]=\left[\begin{array}{ccccc} \lambda_{11} & \cdots & \lambda_{i1} & \cdots & \lambda_{n1}\\ \vdots & \ddots & \vdots & & \vdots \\ \lambda_{1j} & & \lambda_{ij} & & \lambda_{nj}\\ \vdots & & \vdots & \ddots & \vdots \\ \lambda_{1m} & \cdots & \lambda_{im} & \cdots & \lambda_{nm} \end{array}\right]\left[\begin{array}{c} u_{1}\\ \vdots \\ u_{i}\\ \vdots \\ u_{n} \end{array}\right]$$

(9) s=V⋅u

where *s* is the vector of surface deformation of the integrated subreflector, *V* is the actuator’s influence matrix, and *u* is the vector of actuator’s adjustment length. Equation (9) is the SAF of the integrated subreflector.

As shown in Figure 1, the surface deformation of the subreflector will cause the wavefront error, and it can be determined by Equation (10). The equation describes the relationship between the subreflector shape deformation and the wavefront error $w_{R}$ (wave path difference).

(10) $$w_{R}=2\delta_{n}\cdot q$$

where $\delta_{n}$ is the normal component of the subreflector surface displacement, which is the surface deformation caused by the actuator’s adjustment, and *q* is the cosine of the normal direction of each discrete point of the subreflector. The wavefront error control function (WECF) (10) can be expressed by

(11) $$w_{R}=2V\cdot u\cdot q$$

### 3.2. Least Squares Matrix Transformation

We will adopt the method of the minimum residual wave path difference to obtain the subreflector actuator’s length. It is assumed that the active subreflector is controlled by *N* actuators, and the motion length of actuators is adjusted to minimize the wave path difference, and it can be given by

(12) $$e=\left\|w_{R}(x,y)-w_{T}(x,y)\right\|=\min$$

where $w_{T}$ is the wavefront error before the shape is corrected, $w_{R}$ is the wave path difference caused by the actuator, *e* is the residual wavefront error after the shape is corrected, and ‖·‖ denotes the corrected error sum of squares. Then, the Equation (12) can be written as

(13) $$e=\left\|w_{R}(x,y)-w_{T}(x,y)\right\|=\sum_{j=1}^{m}{\left[w_{R}(x_{j},y_{j})-w_{T}(x_{j},y_{j})\right]}^{2}$$

where *m* is the number of discrete points on the subreflector and *j* is the *j*th point, $p_{j}=\left(x_{j},y_{j}\right)$.

To obtain the optimal actuators adjustment amount, the Equation (12) can be solved by

(14) $$\frac{\partial e}{\partial u_{i}}=\frac{\partial }{\partial u_{i}}\sum_{j=1}^{m}{\left[w_{R}\left(p_{j}\right)-w_{T}\left(p_{j}\right)\right]}^{2}=\sum_{j=1}^{m}\left[2w_{R}\frac{\partial w_{R}}{\partial u_{i}}-2w_{T}\frac{\partial w_{R}}{\partial u_{i}}\right]=0$$

(15) $$\sum_{j=1}^{m}\left\{\left[\sum_{i=1}^{n}2u_{i}\lambda_{i}(p_{j})q(p_{j})\right]\lambda_{i}(p_{j})q(p_{j})\right\}=\sum_{j=1}^{m}w_{T}(p_{j})\lambda_{i}(p_{j})q(p_{j})\;\;i=1,\ldots ,n$$

and the equations set can be written as

(16) $$\left\{ \begin{array}{l} 2u_{1}\sum_{j=1}^{m}\lambda_{1}(p_{j})\cdot \lambda_{1}(p_{j})\cdot q(p_{j})+\ldots +2u_{n}\sum_{j=1}^{m}\lambda_{n}(p_{j})\cdot \lambda_{1}(p_{j})\cdot q(p_{j})=\sum_{j=1}^{m}w_{T}(p_{j})\cdot \lambda_{1}(p_{j})\cdot q(p_{j})\\ \begin{array}{ccc} \ldots \;\;\;\;\;\;\;\;\; & \ldots \;\;\;\;\;\;\;\;\; & \ldots \end{array}\\ 2u_{1}\sum_{j=1}^{m}\lambda_{1}(p_{j})\cdot \lambda_{N}(p_{j})\cdot q(p_{j})+\ldots +2u_{n}\sum_{j=1}^{m}\lambda_{n}(p_{j})\cdot \lambda_{n}(p_{j})\cdot q(p_{j})=\sum_{j=1}^{m}w_{T}(p_{j})\cdot \lambda_{n}(p_{j})\cdot q(p_{j}) \end{array}\right.\;$$

The matrix form is:

(17) $$\left[\begin{array}{cccc} 2\sum \lambda_{1}\cdot \lambda_{1}\cdot q_{1} & 2\sum \lambda_{2}\cdot \lambda_{1}\cdot q_{1} & \cdots & 2\sum \lambda_{N}\cdot \lambda_{1}\cdot q_{1}\\ 2\sum \lambda_{1}\cdot \lambda_{2}\cdot q_{2} & 2\sum \lambda_{2}\cdot \lambda_{2}\cdot q_{2} & \cdots & 2\sum \lambda_{N}\cdot \lambda_{2}\cdot q_{2}\\ \vdots & \vdots & \ddots & \vdots \\ 2\sum \lambda_{1}\cdot \lambda_{N}\cdot q_{n} & 2\sum \lambda_{2}\cdot \lambda_{n}\cdot q_{n} & \cdots & 2\sum \lambda_{n}\cdot \lambda_{n}\cdot q_{n} \end{array}\right]\cdot \left[\begin{array}{c} u_{1}\\ u_{2}\\ \vdots \\ u_{n} \end{array}\right]=\left[\begin{array}{c} \sum w_{T}\cdot \lambda_{1}\cdot q_{1}\\ \sum w_{T}\cdot \lambda_{2}\cdot q_{2}\\ \vdots \\ \sum w_{T}\cdot \lambda_{n}\cdot q_{n} \end{array}\right]$$

(18) C⋅u=b

where u is the adjustment of the actuators, *C* is the coefficient matrix, and *b* is the minimum value of the residual error. In order to obtain the optimal adjustment, the least square method can be used to construct the 2-norm of the minimization residual equation and its minimum value can be obtained by

(19) e(u)=b−Cu

(20) $$e_{1}\left(u\right)={\left\|e(u)\right\|}_{2}=e^{T}e=b^{T}b-2u^{T}C^{T}b+u^{T}C^{T}Cu=\min$$

(21) $$\frac{\partial e_{1}(u)}{\partial u}=-2C^{T}b+2C^{T}Cu=0$$

where *e*_1_(*u*) is the residual error. The optimal equation can be expressed as

(22) $$C^{T}Cu=C^{T}b$$

The solution of the linear equations is the optimal adjustment displacement of the actuator required for the modification of the subreflector’s active surface shape. The solution can be expressed by

(23) $$u={\left(C^{T}C\right)}^{-1}C^{T}b$$

In summary, the above method can be used to obtain the adjustment amount of the subreflector actuators, allowing for further correction of the residual wavefront error after adjusting the subreflector pose. 

## 4. The Analysis and Results

In this section, the validity of the method will be verified by simulation experiments. The initial assumption is that the position adjustment of the subreflector has partially corrected the structural deformation of the primary reflector caused by the gravity load. Subsequently, the wavefront residual minimization adjustment method will be employed to correct the residual wavefront error by adjusting the shape of the subreflector. 

**Figure 6:**
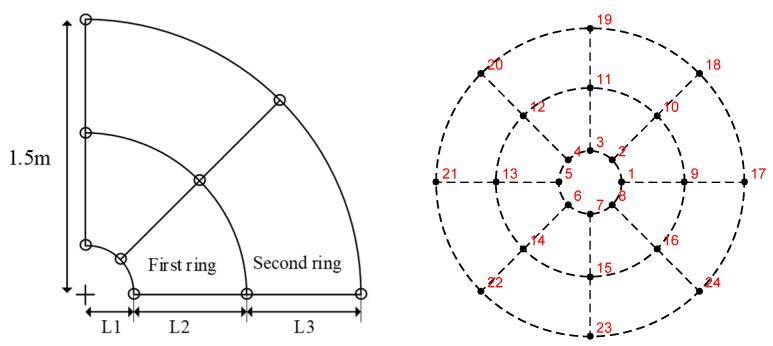
The distribution diagram of 3 m subreflector actuators (L1 = 0.3 m, L2 = 0.6 m, L3 = 0.6 m).

The experimental object for this study is a 25 m Cassegrain antenna with a focal ratio of 0.3 and a subreflector with a 3 m diameter. It is assumed that the integrated subreflector is used, and the actuators are divided into three rings along the radial direction. Figure 6 shows the arrangement of the 24 actuators, which are symmetrically distributed in the circumferential rotation. 

**Figure 7:**
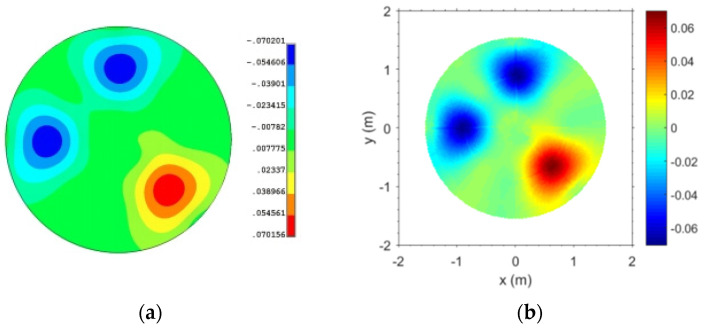
Subreflector deformation distribution. (**a**) Finite element analysis; (**b**) Interpolation fitting.

The weighted index *μ* in Equation (5) was obtained by the finite element analysis. Then, the optimal parameter μ* was obtained by using a sequential quadratic programming algorithm to optimize the target of minimum residual difference between the plane displacement obtained by the interpolation function and the finite element analysis result. Through calculation, the optimal parameter μ* = 1.70. As shown in Figure 7, the subreflector deformation distribution was fitted, and the RMS (Root Mean Square) is 0.47 × 10^−4^. 

**Figure 8:**
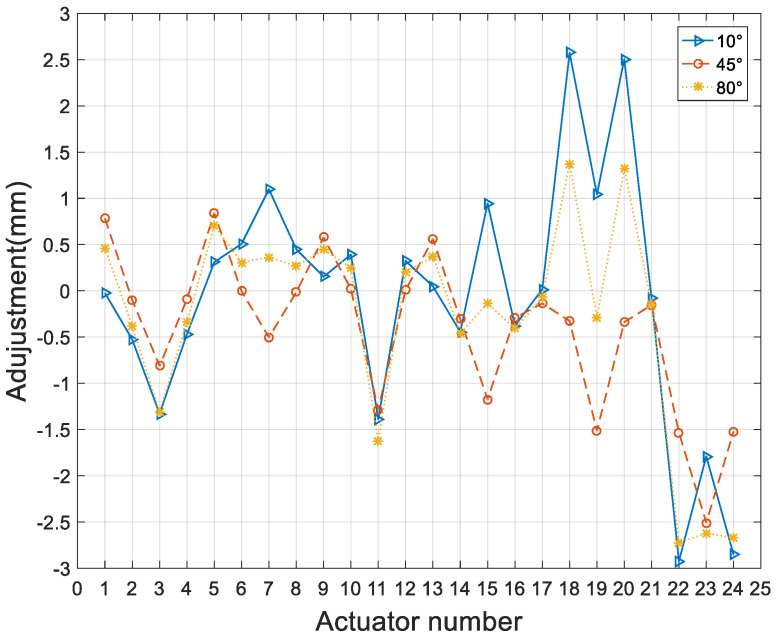
The adjustment amount of subreflector actuators.

For the wavefront errors of 10°, 45°, and 80° elevation angles, we adjusted the position and pose of the subreflector and then used the method of minimizing the residual error to determine the adjustment amount of the subreflector actuator. The inverse distance weighting coefficient of the corresponding points was calculated using Equation (5), and then the residual wavefront error *W_T_* was calculated using Equation (18) to obtain the matrix *C* and *b*, and then the adjustment amount of the actuator was obtained by Equation (23) according to the distribution position of the actuators and the location of the sampling point of the port data. The adjustment quantity of actuators was shown in Figure 8 for the 10°, 45°, and 80° elevation angles. It was seen from the figure that the range of actuators adjustment quantity of the subreflector was −3 mm~ +3 mm; most of the actuators had little motion, |*u*| < 1 mm.

**Figure 9:**
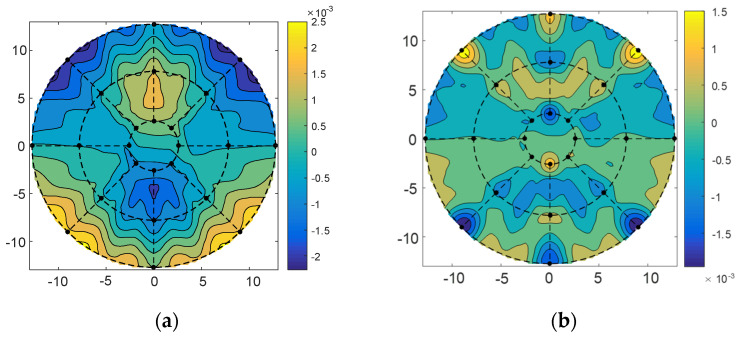
(**a**) The distribution before correction for wavefront error of 10°; (**b**) The distribution after correction for wavefront error of 10°.

**Figure 10:**
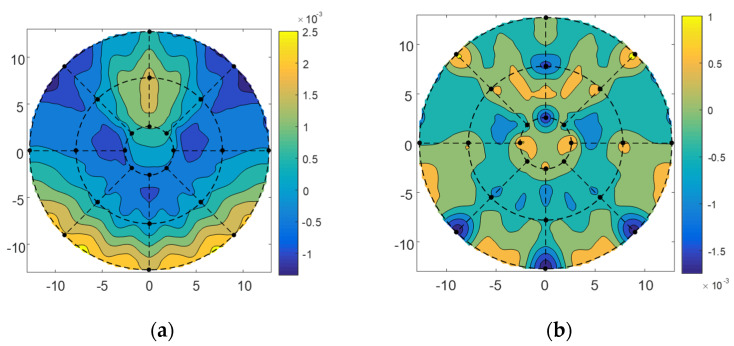
(**a**) The distribution before correction for wavefront error of 45°; (**b**) The distribution after correction for wavefront error of 45°.

**Figure 11:**
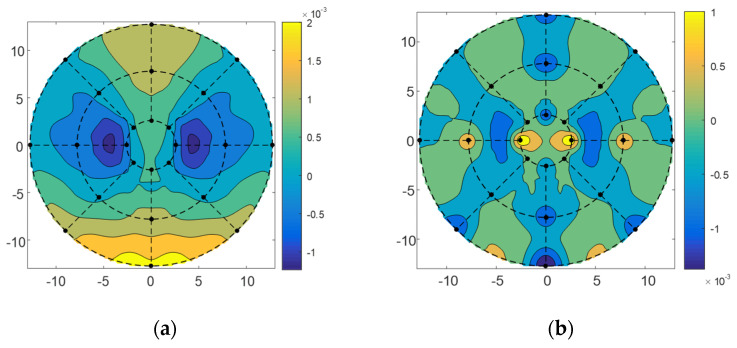
(**a**) The distribution before correction for wavefront error of 80°; (**b**) The distribution after correction for wavefront error of 80°.

**Figure 12:**
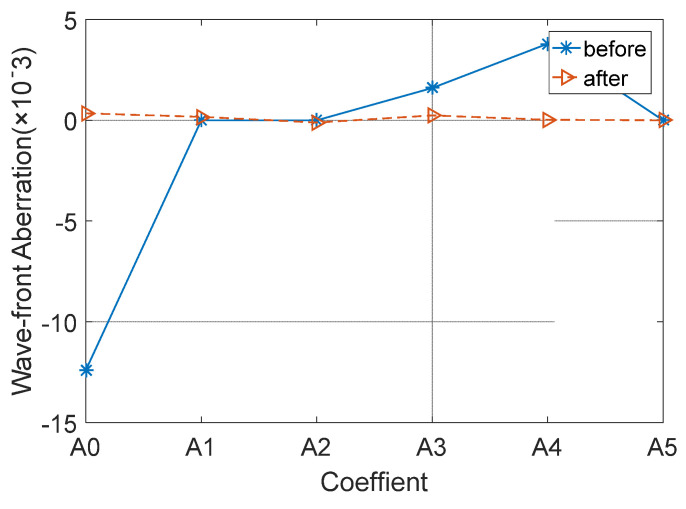
Correction for wavefront aberration at 10° elevation angle.

**Figure 13:**
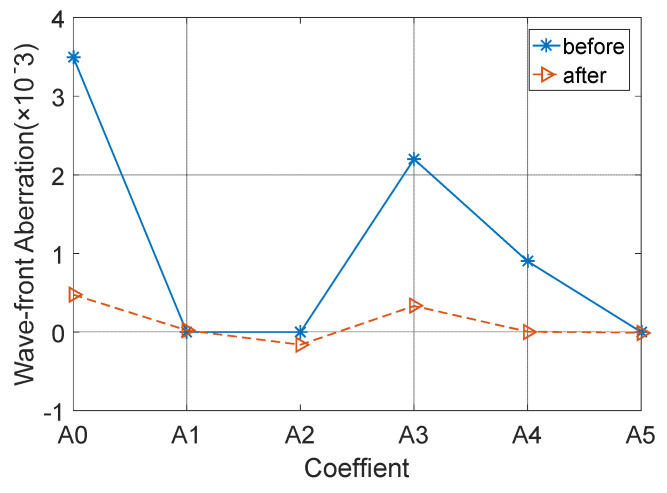
Correction for wavefront aberration at 45° elevation angle.

**Figure 14:**
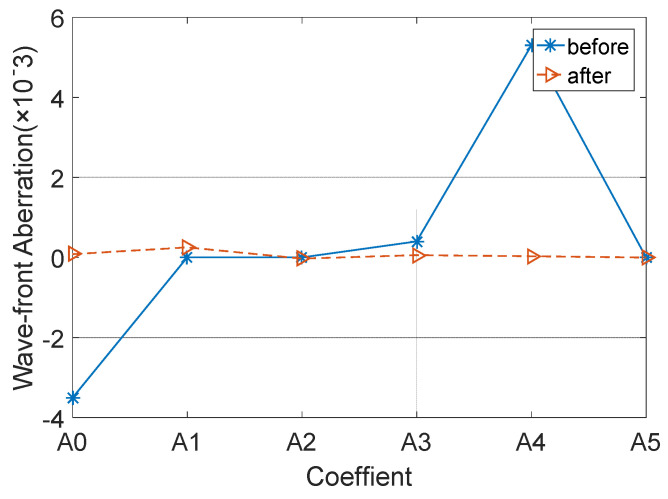
Correction for wavefront aberration at 80° elevation angle.

The subreflector shape was adjusted based on the amount of adjustment for each actuator. Figure 9, Figure 10 and Figure 11 show the distribution of wavefront errors at 10°, 45°, and 80° with the subreflector shape adjustment. The corrected residual surface errors were analyzed for aberrations, and the resulting aberration coefficients of wavefront errors are shown in Figure 12, Figure 13 and Figure 14. The coefficients include A0, A1, A2, A3, A4, and A5, which represent different aberration features: piston, tilt, defocus, astigmatism, coma, and spherical.

As shown in Figure 9, Figure 10 and Figure 11, The adjustment of the subreflector shape effectively corrected the residual wavefront errors, primarily small-scale errors. This correction resulted in decreased RMS at various elevation angles. So, it can be inferred that the comprehensive performance of the subreflector is improved. As shown in Figure 12, Figure 13 and Figure 14, the residual primary aberration coefficients (mainly astigmatism A3 and coma A4) were reduced greatly after the subreflector shape was adjusted. In particular, the coma coefficient A4 was reduced to zero, and the astigmatism coefficient A3 was near zero. It is indicated that the primary aberration has been effectively corrected.

## 5. Conclusions

In this paper, a new method of subreflector adjustment with residual wavefront error elimination is presented. The characteristic of wavefront error caused by the subreflector shape of dual-reflector antenna has been studied, and the relationship between the adjustment of the subreflector actuators and the wavefront errors is derived. The wavefront error control function is constructed based on the analysis of the wave path difference and the surface deformation of the subreflector, utilizing the error influence matrix. By solving the matrix transformation using the least squares method, the optimal adjustment amount of subreflector actuators is determined, allowing for rapid adjustment. Simulation experiments demonstrate that after adjusting the surface shape of the subreflector according to the calculated adjustment length of actuators, effective correction of wavefront aberrations such as astigmatism and coma is achieved, resulting in improved antenna performance.
